# Modeling and Relative Permittivity Modulation of Cu/PDMS Capacitive Flexible Sensor for Pressure Sensing

**DOI:** 10.3390/s25030637

**Published:** 2025-01-22

**Authors:** Xu Wang, Yuelong Zhang, Tian Zhang, Guanyu Fu, Yinlong Zhu, Ying Liu

**Affiliations:** 1 College of Mechanical and Electronic Engineering, Nanjing Forestry University, Nanjing 210037, China; zhangyl@njfu.edu.cn (Y.Z.); zhangtian202501@163.com (T.Z.); fuguanyuop@njfu.edu.cn (G.F.); ylzhu@njfu.edu.cn (Y.Z.); liuying@njfu.edu.cn (Y.L.); 2 State Key Laboratory of Robotics, Shenyang Institute of Automation, Chinese Academy of Sciences, Shenyang 110169, China

**Keywords:** capacitive, tactile sensor, flexible sensor, relative permittivity modulation

## Abstract

This study aims to establish an equivalent parallel capacitance model for a copper/polydimethylsiloxane (Cu/PDMS) capacitive flexible pressure sensor and modulate its relative permittivity to optimize pressure sensing performance. The Cu/PDMS composite material is an ideal dielectric layer for sensors due to its high dielectric constant and tunable elasticity. By adjusting the different mixing ratios of PDMS and copper particles in micro size, the components and structure properties of the composite material can be modified, thereby affecting the electrical and mechanical performance of the sensor. We used finite element analysis (FEA) to model the sensor structure and studied the capacitance changes under various normal loading conditions to assess its sensitivity and distribution characteristics. Experimental results show that the sensor has good sensitivity and repeatability in the pressure range of 0 to 50 kPa. Additionally, we explored the effect of the addition of carbon black particles. It could be inferred that the added carbon black can enhance electrical properties due to its conductivity, which would be consequenced by the distribution optimization of Cu particles for carbon black’s low density, and it can mechanically restore some flexibility up to nearly 20%. Through these studies, our work can provide theoretical support for the design and application of flexible pressure sensors.

## 1. Introduction

With the continuous advancement of robotics technology and the expansion of its application fields, the demand for enhancing robot performance to meet higher operational standards is growing [1,2]. Especially in human–robot interaction, tactile perception, as a fundamental form of information interaction, has become a top research priority [3,4,5]. To enable robots to more accurately perceive and respond to the complex changes in the working environment, scholars have proposed integrating flexible pressure sensors into the robot system to enhance its environmental adaptability and interactive capabilities. Flexible pressure sensors, which are characterized by their small size, low weight, and high flexibility, have been proven to effectively enhance the functionality and practicality of robots in various scenarios, including special environment operations, medical assistive devices, and human–robot interaction [6,7,8,9]. Among the types of flexible pressure sensors, capacitive sensors have attracted widespread attention in recent years due to their excellent dynamic response, simple structure, low power consumption, high stability, and cost-effectiveness [10,11,12]. These sensors have shown great potential in applications such as precise robot grasping, human function monitoring, and rehabilitation medicine [13,14,15,16]. Despite this, existing capacitive flexible sensors still face many challenges in improving sensitivity and addressing size coupling issues, which limit their performance and scope of application. Therefore, further in-depth research and the optimization of the sensitivity and overall functionality of these sensors are the current research focus [17].

The performance of flexible pressure sensors can be assessed through several fundamental parameters, including pressure sensitivity, detection range, linearity, response time, recovery time, and durability. Among these parameters, sensitivity is particularly important because it directly reflects the sensor’s ability to sense changes in external pressure [18,19]. When the sensor is subjected to a load, the dielectric layer is prone to significant strain, which is key to improving sensor sensitivity [20,21,22]. By designing the surface microstructure of the dielectric layer, such as introducing microdomes, pyramidal structures, or by fabricating the dielectric material into porous or composite forms, the performance of capacitive flexible pressure sensors can be greatly enhanced [23]. These microstructures or complex compositions of the dielectric layer not only enhance the material’s deformation capability, thereby increasing sensitivity, but may also improve the sensor’s linearity and dynamic response characteristics [24,25,26].

Mannsfeld et al. [27] improved the sensitivity of a device by 30 times after designing a pyramid-shaped microstructure on the surface of the dielectric layer. Zhou et al. [28], inspired by human hair skin, developed a flexible capacitive pressure sensor with a dielectric layer composed of a hair-like microfibril array (MCA) made of PDMS and carbon-based iron particles. The surface microstructure of the dielectric layer can be adjusted by the magnetic field generated by a portable magnet, thereby enabling the sensor to have high sensitivity (0.28 kPa^−1^ in the 0–10 kPa range), a wide detection range (0–200 kPa), a low detection limit (2 Pa), and good structural robustness and performance stability. Kwon et al. [29] reported a flexible wearable pressure sensor based on the giant piezo capacitance effect of a three-dimensional microporous dielectric elastomer (Ecoflex), which can exhibit highly sensitive and stable pressure sensing behavior over a large tactile pressure range, with a sensitivity of 0.601 kPa^−1^ below 5 kPa and no significant decrease in sensitivity over a wide dynamic range (0.1 Pa–130 kPa).

The flexible sensor can be applied as an electronic skin to determine the grip force level of mechanical fingers and as a bandage-type pressure sensing device to detect pulse signals from a person’s wrist [30,31,32]. When optimizing the performance of capacitive flexible pressure sensors, researchers often face a common problem, which is that increasing sensitivity often sacrifices the width of the detection range. This limiting relationship poses a significant challenge in the design of sensors. In addition, capacitive flexible pressure sensors also face several other key issues in practical applications, including hysteresis effects, insufficient stability, and the optimization demand for arraying [33,34,35]. Hysteresis effects can affect the repeatability and predictability of sensor outputs, while stability issues limit their reliability under long-term or extreme conditions. In terms of arraying, although it can improve spatial resolution, the increased complexity in design and integration also brings additional burdens to the actual deployment and maintenance of the system.

Capacitive flexible pressure sensors have gained significant attention in recent years due to their high sensitivity, flexibility, and potential applications in wearable electronics and soft robotics. In this study, we propose a novel equivalent parallel capacitance model for a copper/polydimethylsiloxane (Cu/PDMS) capacitive pressure sensor. The model focuses on modulating the relative permittivity of the composite material to enhance its pressure sensing performance. To achieve this, we investigate the use of conductive fillers, including copper (Cu) powder and carbon black (CB) particles, to improve the dielectric properties of the Cu/PDMS composite. Our work also examines the role of the percolation threshold, where the conductive fillers form continuous conductive paths, significantly improving the electrical conductivity and performance of the sensor. Moreover, the addition of carbon black particles not only boosts the viscosity of the composite but also enhances the dispersion of Cu powder within the PDMS matrix, leading to optimized dielectric properties. This comprehensive approach provides a deeper understanding of the material’s behavior and offers valuable insights for designing more efficient capacitive pressure sensors. In our study, we have compared the proposed capacitive array sensor with several recent works in the field. Specifically, we refer to the work by Gong et al. [14], which focuses on a soft capacitive tactile sensor with a carbon black/PDMS composite dielectric layer for multi-directional force sensing. Our results show significant improvements in both the pressure detection range and capacitance retention rate. The pressure detection range is 0–50 kPa, whereas Gong et al. reported a range of 0–10 kPa.

## 2. Working Principle of Capacitive Tactile Sensors

### 2.1. Structure of the Sensor

**Figure 1:**
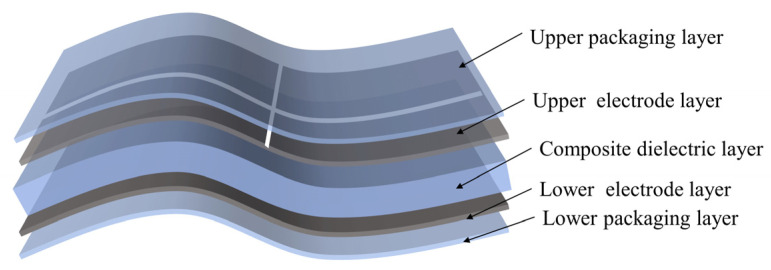
Capacitive sensor structure diagram.

This section describes the design of a flexible capacitive sensor, the structure of which is shown in Figure 1. The sensor mainly consists of five layers of materials, namely the upper encapsulation layer, the upper electrode layer, the composite dielectric layer, the lower electrode layer, and the lower encapsulation layer. The composite dielectric layer is placed between the upper and lower electrode layers, with dimensions of 11 mm × 11 mm × 1 mm, to ensure it has appropriate dielectric properties and mechanical stability. Both the upper and lower electrode layers are made of conductive fabric, each with a thickness of 0.1 mm. The upper electrode layer is designed to consist of four identical square electrodes, each with a size of 5 mm × 5 mm, while the lower electrode layer forms a complete square with dimensions of 11 mm × 11 mm. Regarding the design choice of the upper electrode layer, we did consider adjusting the dimensions of the four square electrodes to 5.5 mm × 5.5 mm in order to make the total area match the lower electrode layer (11 mm × 11 mm). However, this would have resulted in the four electrodes being directly connected, causing significant interference between them. When the electrodes are connected, it leads to a breakdown of the intended electrical field distribution, as the proximity between the electrodes causes mutual interference. This interference would disrupt the sensor’s functionality, as the electrical signals would overlap and distort, preventing the sensor from operating properly. For this reason, we deliberately chose to keep a gap between the electrodes to ensure optimal performance by maintaining distinct electric fields for each electrode, which enhances the sensor’s accuracy and stability. The upper and lower encapsulation layers both use PDMS material with dimensions of 11 mm × 11 mm × 0.1 mm, which not only provides necessary physical protection but also ensures the flexibility and durability of the sensor. This multi-layer structural design helps to enhance the testing stability and sensitivity of the sensor, making it particularly suitable for applications requiring high-precision pressure mapping.

In the manufacturing of flexible sensors, common dielectric layer materials include polyimide (PI), polyethylene naphthalate (PEN), polyethylene terephthalate (PET), polydimethylsiloxane (PDMS), silicone rubber, and polyvinyl alcohol (PVA), among others. Among them, polydimethylsiloxane (PDMS) is widely used in various flexible sensors due to its excellent biocompatibility, flexibility, and stability, as well as the simplicity and low cost of its preparation process. Based on these advantages, this article selects PDMS as the base material for the dielectric layer in the preparation of flexible sensors.

When selecting electrode materials suitable for flexible sensors, although traditional metals such as gold, silver, copper, and zinc possess excellent electrical properties, they often lack the flexibility required for flexible sensors. In contrast, organic conductive materials, such as carbon nanotubes, carbon black, and graphene, show good electrical conductivity but face significant technical challenges in their fabrication process. Therefore, this study ultimately chose conductive fabric, which is easily obtainable and processable, as the electrode material. Conductive fabric not only has superior mechanical compatibility but has also shown excellent performance in wearable technology and other flexible electronic devices, meeting the performance requirements of sensors in a variety of usage environments.

### 2.2. Working Principle of Normal Pressure

Flexible capacitive sensors operate based on the working principle of capacitors, converting non-electrical quantities into capacitance to detect and feedback input signals. Flexible capacitive sensors typically adopt the structure of a parallel-plate capacitor, which includes two parallel electrode plates and a flexible dielectric layer sandwiched between them. Ignoring the edge effects, the capacitance *C* of the flexible capacitive sensor [14] is given by

(1) $$C=\frac{\varepsilon_{r}\varepsilon_{0}S}{d}$$

in which $\varepsilon_{r}$ is the relative permittivity of dielectric, $\varepsilon_{0}$ is the permittivity of free space (8.854 × 10^−12^ F/m), *S* is the projected area of both electrodes, and *d* is the distance between the two electrode plates.

**Figure 2:**
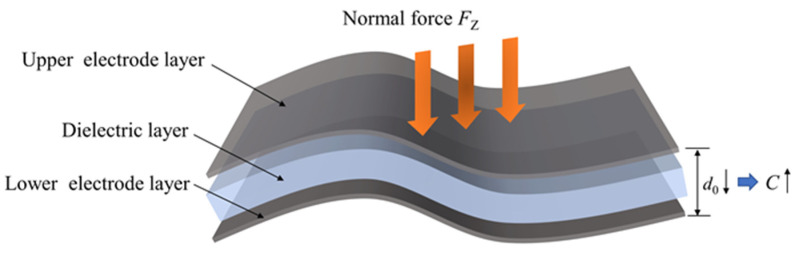
Diagram of normal force.

As shown in Figure 2, under the action of a normal force, the thickness of the dielectric layer in the flexible capacitive sensor changes, which in turn alters the distance *d* between the upper and lower electrodes, thereby causing a change in the capacitance value.

Given that the normal force applied to the sensor is *F*_z_, the initial capacitance value is $C_{0}$, the initial projected area between the upper and lower electrodes is $S_{0}$, and the initial distance is $d_{0}$, after the sensor is subjected to the normal force, the capacitance value becomes *C*, the projected area between the upper and lower electrodes remains *S* (i.e., $S_{0}$ = *S*), and the distance becomes *d*, with the change in distance being Δ*d*; then, the change in capacitance value of the flexible sensor, $\Delta C_{Z}$, [14] is

(2) $$\Delta C_{Z}=C-C_{0}=\frac{\varepsilon \varepsilon_{0}S_{0}}{d}-\frac{\varepsilon \varepsilon_{0}S}{d_{0}}=\frac{\varepsilon \varepsilon_{0}S_{0}\Delta d}{dd_{0}}$$

The change in distance between the upper electrode and the lower electrode Δd can be expressed as

(3) $$\Delta d=d_{0}-d$$

The relationship between the change in capacitance of the flexible sensor and the change in spacing [14,35] is

(4) $$\frac{\Delta C_{Z}}{C_{0}}=\frac{\Delta d}{d_{0}-\Delta d}$$

Through subsequent experiments and the relevant literature [36,37], it has been found that doping conductive fillers in the dielectric layer of flexible capacitive sensors can effectively increase the relative permittivity of the dielectric layer. The change in the relative permittivity $\varepsilon_{r}$ can reflect the degree of internal polarization of the material and affect the change in capacitance, thereby revealing the impact of conductive particle doping on the performance of flexible capacitive sensors. The relative permittivity $\varepsilon_{r}$ of the dielectric layer can be calculated by transforming Equation (1):

(5) $$\varepsilon_{r}=\frac{C\cdot d}{\varepsilon_{0}\cdot S}$$

The trend of relative permittivity changes in the flexible capacitive dielectric layer system doped with conductive fillers can be explained by percolation theory [38,39], and its mathematical expression is as follows:

(6) $$\varepsilon_{r}\propto {\left(P_{c}-P\right)}^{-t},P\leq P_{c}$$

where $\varepsilon_{r}$ represents the dielectric constant of the composite dielectric layer, *P* is the doping content of conductive fillers, $P_{c}$ is the percolation threshold of conductive fillers, and *t* is the critical exponent of the dielectric region, approximately 0.64. From the formula, it can be seen that when the doping content of conductive fillers approaches the percolation threshold, the dielectric constant of the composite dielectric layer will be significantly increased. However, when the filler content exceeds the percolation threshold, the dielectric constant of the composite material is inversely proportional to the filler content. Therefore, it is necessary to control the content of conductive fillers below the percolation threshold to obtain the optimal dielectric constant value, thereby optimizing the performance of the sensor.

**Figure 3:**
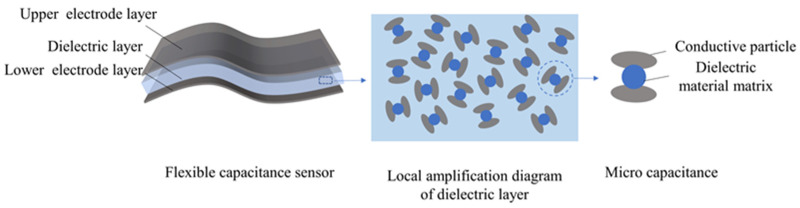
Schematic distribution of conductive particles in the dielectric layer.

In this experiment, spherical conductive particles (copper powder) with a diameter of 3 μm were selected as conductive fillers. During the actual doping process, these conductive particles can be uniformly dispersed throughout the dielectric layer. As shown in Figure 3, the conductive particles come into contact with the dielectric matrix, forming micro-capacitive structures. These micro-capacitors are integrated together through a parallel effect, thereby changing the relative permittivity of the entire dielectric layer. By adjusting the doping ratio of conductive particles, the dielectric properties of the dielectric layer can be effectively regulated, thereby optimizing the sensing characteristics of the capacitive device.

**Figure 4:**
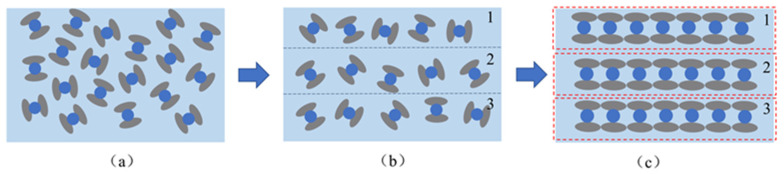
Diagram of the formation process of the composite dielectric layer. (**a**) Local schematic diagram of dielectric layer. (**b**) Dielectric layer of multilayer structure. (**c**) Composite dielectric layer structure.

**Figure 5:**
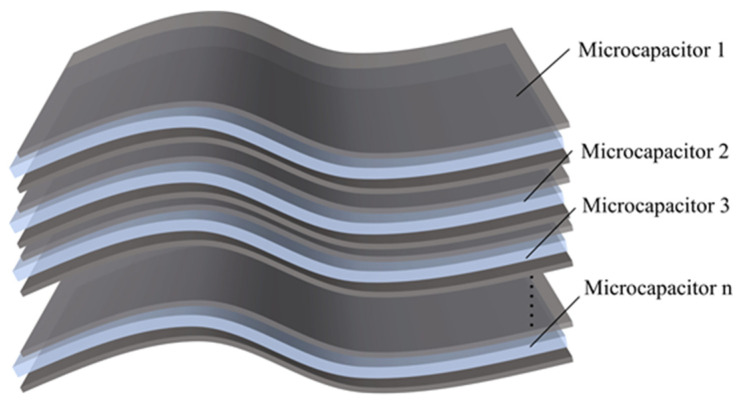
Schematic modeling of a composite dielectric layer.

To further explore the mechanism by which the doping of conductive fillers affects the change in the relative permittivity of the flexible dielectric layer, it is assumed that the conductive fillers are uniformly distributed throughout the flexible dielectric layer. The dielectric layer is conceptualized as a multi-layered structure, as shown in Figure 4b. When the conductive fillers are filled to a certain density in a certain layer of the dielectric layer, that layer can be regarded as a new micro-capacitor. Each layer of micro-capacitors is connected in parallel, thus forming the composite dielectric layer, as shown in Figure 4c. To more clearly describe this process, Figure 5 shows a schematic diagram of the composite dielectric layer model. In the design of micro-capacitors, the effects of series and parallel configurations on the total capacitance are significantly different. In the serial model, the capacitance value of the sensor would remain the same as that of the undoped conductive particles, which would not capture the performance enhancement induced by the doping. In the parallel model, the capacitance value of the flexible sensor doped with conductive fillers becomes n2 times that of the undoped conductive particles. The parallel configuration is therefore more appropriate for explaining the performance improvements observed in the sensor due to the doping of conductive fillers in the dielectric layer.

After doping with conductive fillers, the composite dielectric layer is equivalent to breaking down the original sensor into *n* micro-capacitors with a thickness of *d*/*n* connected in parallel. At this point, the equivalent capacitance value *C*′ of the sensor is

(7) $$C^{\prime }=\frac{\varepsilon_{r}\varepsilon_{0}S}{\frac{d}{n}}\times n=n^{2}\times C$$

In this hypothetical model, the capacitance value of the flexible sensor doped with conductive fillers becomes *n*^2^ times that of the undoped conductive particles. This model provides an intuitive explanation for the percolation phenomenon caused by the doping of conductive fillers in the dielectric layer. Specifically, when the mass fraction of conductive particles reaches the percolation threshold, these particles form a continuous conductive path within the dielectric layer, turning the original dielectric layer into multiple micro-capacitors in parallel, thereby significantly enhancing the electrical properties of the dielectric layer.

## 3. Preparation of the Soft Capacitive Sensor

### 3.1. Fabrication and Testing of Dielectric Layers

In this experiment, to enhance the relative dielectric constant of the dielectric layer of the flexible capacitive sensor, copper powder with a particle size of about 3 μm was selected as the dopant. First, the copper powder was pre-treated by placing an appropriate amount of copper powder into a beaker containing 1/3 of its capacity of anhydrous ethanol and then placing the beaker into an ultrasonic cleaner for 30 min at room temperature to clean the surface dust of the copper particles; after the cleaning, the copper was taken out and placed in a hot air drying oven at 50 °C for 1 h to ensure that the residual anhydrous ethanol was completely volatilized; finally, the dried copper powder was ground in a mortar to ensure that the copper particles were fully dispersed and to prevent adhesion between the particles.

**Figure 6:**
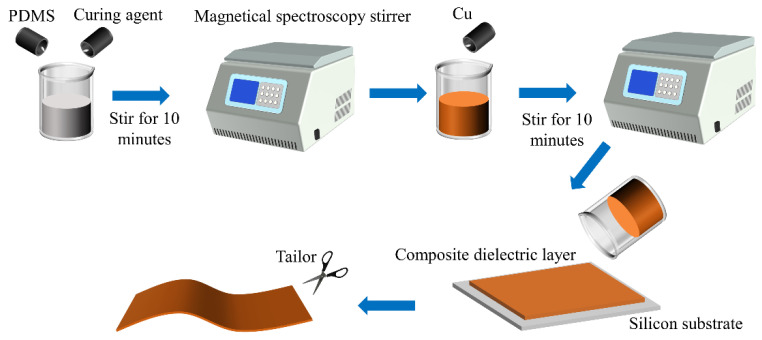
Schematic diagram of Cu/PDMS composite dielectric layer preparation.

As shown in Figure 6, this experiment prepared Cu/PDMS composite dielectric layers with different copper (Cu) contents, with the mass ratios of Cu being 0 wt%, 2 wt%, 6 wt%, 10 wt%, 14 wt%, 20 wt%, 25 wt%, 30 wt%, 35 wt%, 38 wt%, 40 wt%, 42 wt%, 45 wt%, and 50 wt%. When the mass fraction of copper powder is 0 wt%, the dielectric layer consists solely of PDMS material. Under this condition, the measured relative dielectric constant (*ε*_r_) of PDMS can serve as a reference baseline for subsequent measurements of the relative dielectric constant of composite materials containing copper powder. Since the relative density of PDMS is approximately 1 g/mL and the density of copper is about 8.960 g/cm^3^, there is a difference of nearly nine times in density. If the mass ratio of Cu is further increased, the main properties of the material will become copper-based, which is not helpful for the purpose of the experiment. Therefore, the content of Cu is limited to below 50%.

During the experimental process, the total mass of PDMS was first fixed, and the required mass ratio of Cu was calculated based on this. After pre-treating the Cu, it was weighed using a balance, and then the pre-polymer PDMS was mixed with the curing agent in a mass ratio of 10:1. This mixture was placed in a vacuum magnetic stirrer and stirred for 10 min to ensure thorough mixing and to remove air bubbles. Subsequently, the Cu powder was added to the pre-mixed PDMS, and the mixture was placed in the vacuum magnetic stirrer again for 10 min to ensure that the Cu was completely and evenly dispersed. Finally, the mixed Cu/PDMS mixture was poured onto the silicon substrate, and the smoothness of the silicon substrate surface and the surface tension of the mixture allowed the mixture to spread smoothly without overflowing the boundaries. The curing process was carried out in a hot air dryer, with a drying temperature set to 70 °C for a duration of 2 h. The cured material was then cut to the required dimensions and used as the dielectric layer for flexible capacitive sensors.

**Figure 7:**
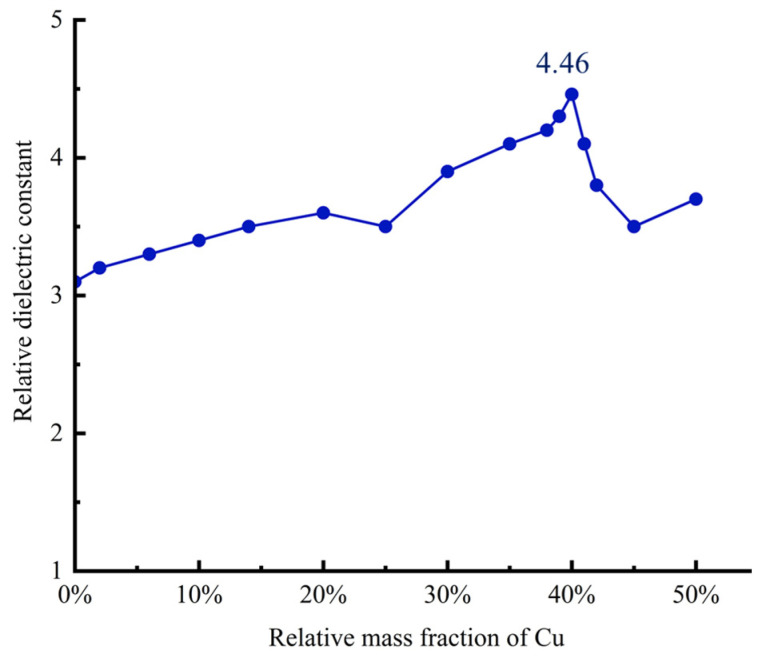
Dielectric constant of carbon black/PDMS composites with different mass fractions.

In this experiment, a high-precision LCR bridge was used to measure the relative dielectric constant $\varepsilon_{r}$ of the dielectric layer of flexible sensors with different mass fractions of copper powder. First, parallel plate electrodes were attached to the top and bottom surfaces of the sample to ensure that the area between the two electrodes remained constant. The test voltage of the LCR bridge was set to 1 V and the frequency to 100 kHz, and the capacitance of the sample was measured through these parameters. During the test, a push–pull force gauge was used to apply different pressures to the surface of the flexible sensor in the normal force direction, and the capacitance values under different pressures were recorded. To reduce measurement errors and ensure the reliability of the data, the capacitance measurement under each applied normal force condition was repeated five times, and the average value was calculated as the representative capacitance value for that condition. To accurately determine the thickness of the dielectric layer, a small section of the sample was taken and observed and measured using a microscope. The thickness measurement of this section was also repeated five times to reduce measurement errors, and then the average value was taken. Finally, the measured average thickness value and capacitance value were substituted into Equation (5), and the sample’s $\varepsilon_{r}$ was calculated according to the formula, with the test results shown in Figure 7.

As shown in Figure 7, the relative dielectric constant of pure PDMS is 3.14. As the mass fraction of Cu powder in the composite dielectric layer increases from 0 wt% to 20 wt%, the relative dielectric constant of Cu/PDMS rises slowly. When the mass fraction of Cu powder reaches 25 wt%, a sharp increase in the relative dielectric constant of the composite dielectric layer is observed; further increasing the mass fraction of Cu to 40 wt%, the dielectric constant reaches its maximum value of 4.46. At this Cu mass fraction, the characteristic of a percolation threshold is exhibited, indicating that a continuous conductive network has been established between Cu particles. Based on this finding, the Cu/PDMS composite dielectric layer containing 40 wt% Cu is selected as the dielectric layer for the flexible capacitive sensor to achieve higher sensing performance.

### 3.2. Optimization of the Component Ratio of the Dielectric Layer Material

**Figure 8:**
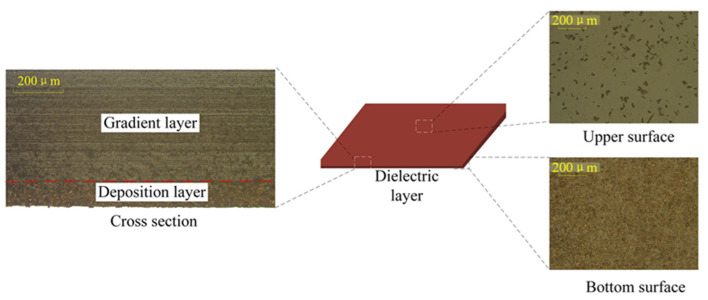
Characterization of 40 wt% Cu/PDMS composite dielectric layer.

**Figure 9:**
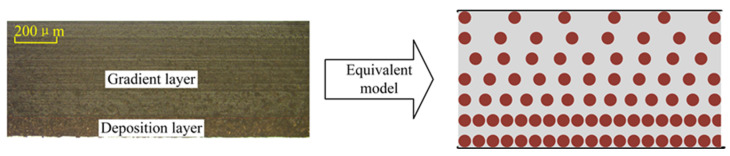
Cu/PDMS cross-sectional view and equivalent model.

Figure 8 presents the characterization results of the surfaces of the Cu/PDMS composite dielectric layer containing 40 wt% Cu. Due to the significant density difference between PDMS and Cu, partial layering of Cu powder occurs during the mixing process. In this composite material, some of the Cu powder is concentrated in the lower layer of PDMS, forming a dense deposit layer, while the other part of the Cu powder is distributed in a step-like pattern within the PDMS, gradually decreasing from top to bottom, forming a step layer. The actual cross-sectional diagram and the equivalent model schematic are shown in Figure 9.

**Figure 10:**
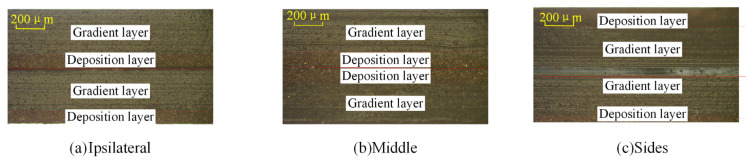
Cross-sectional view of double-layer Cu/PDMS dielectric layer bonding.

To investigate the impact of the distribution of Cu powder in PDMS on the relative dielectric constant of the composite dielectric layer, a set of experiments was designed in this paper. The Cu/PDMS composite dielectric layers with a Cu content of 40 wt% were divided into three different bonding combinations, namely a deposit layer with a step layer, a deposit layer with a deposit layer, and a step layer with a step layer. As shown in Figure 10, the morphologies of the sections of these different combinations after PDMS bonding treatment are displayed in detail.

**Figure 11:**
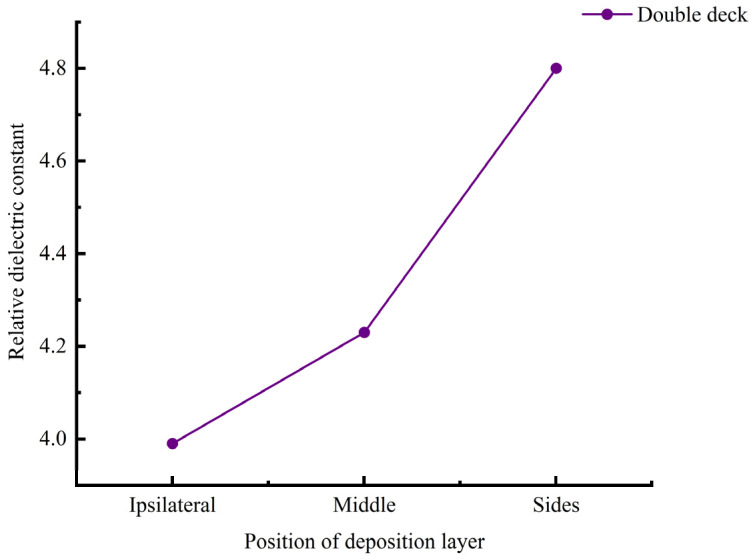
Relative permittivity of different deposition layer locations.

The relative dielectric constants of the three composite dielectric layers with different stratifications after bonding were tested, repeating the aforementioned dielectric constant testing process to ensure the reliability and accuracy of the data. The test results are shown in Figure 11. When the deposit layer is on the same side or in the middle of the two layers of material, it is observed that the relative dielectric constant of the double-layer samples is lower than that of the single-layer samples. Specifically, when the deposit layer is in the middle, its dielectric constant is higher than when the deposit layer is on the same side at both ends. Conversely, when the deposit layer is distributed on both sides of the double layer, its relative dielectric constant is higher than that of the single-layer sample.

Figure 11 shows the significant impact of the distribution of Cu particles in PDMS on the relative dielectric constant of the composite material sensor. Although stirring in a vacuum magnetic stirrer can initially achieve a uniform mixture of Cu and PDMS, due to the significant difference in density between the two, Cu particles inevitably settle during the curing process of the composite material. Therefore, there are two approaches to change the uniform distribution of Cu in PDMS, namely (1) changing the curing time of the Cu/PDMS composite material or (2) changing the viscosity of the Cu/PDMS composite material. Since the composite material is filled with Cu particles and PDMS as the main filling material, the curing time is determined by the properties and thickness of the PDMS material. Although temperature changes can accelerate the curing time of PDMS, Cu particles inevitably cause deposition during the process of pouring the composite material onto the silicon substrate and leveling the composite material on the silicon substrate. Therefore, this paper further explores the addition of carbon black (CB) particles to increase the viscosity of the Cu/PDMS composite material in order to improve the dispersion of Cu particles in the PDMS matrix.

**Figure 12:**
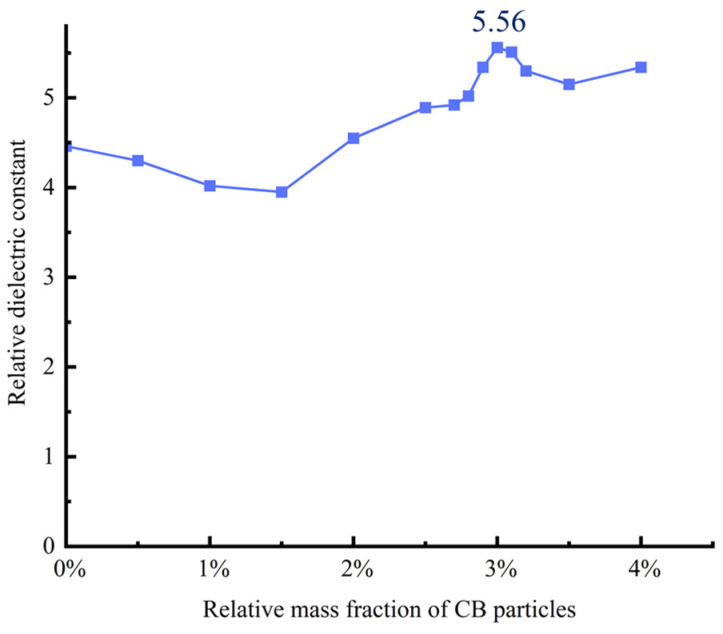
Relative permittivity of different CB doped with 40% Cu.

In the experiment, a Cu/PDMS composite material containing 40 wt% Cu was used, which means the mass ratio of Cu to PDMS is 4:6. On this basis, with PDMS as the filling matrix, different mass fractions of CB conductive particles were added to investigate their impact on the relative dielectric constant of the composite dielectric layer. The addition of CB particles is calculated relative to PDMS, and the amounts are 0 wt%, 0.5 wt%, 1 wt%, 1.5 wt%, 2 wt%, 2.5 wt%, 2.8 wt%, 3 wt%, 3.2 wt%, 3.5 wt%, and 4 wt%. The manufacturing process for each ratio of composite materials is shown in Figure 6, and the corresponding relative dielectric constant test results obtained by LCR measurements are shown in Figure 12.

Under the condition of keeping the dimensions of the dielectric layer consistent, the relative dielectric constant of the Cu/CB/PDMS composite material generally shows an increasing trend with the increase in CB particle content. When the content of CB particles relative to PDMS is 3 wt%, its relative dielectric constant is the highest at 5.56. The experimental data show that when the mass fraction of CB particles relative to PDMS reaches 3 wt%, the dielectric constant reaches its maximum value of 5.56. However, further increasing the content of CB particles will lead to a gradual decrease in the resilience of the composite material. In particular, when the CB content exceeds a certain threshold, the composite material based on PDMS will lose its curing ability. Therefore, the Cu, CB, and PDMS are mixed in a mass ratio of 4:0.42:10 to prepare the dielectric layer for the flexible capacitive sensor.

**Figure 13:**
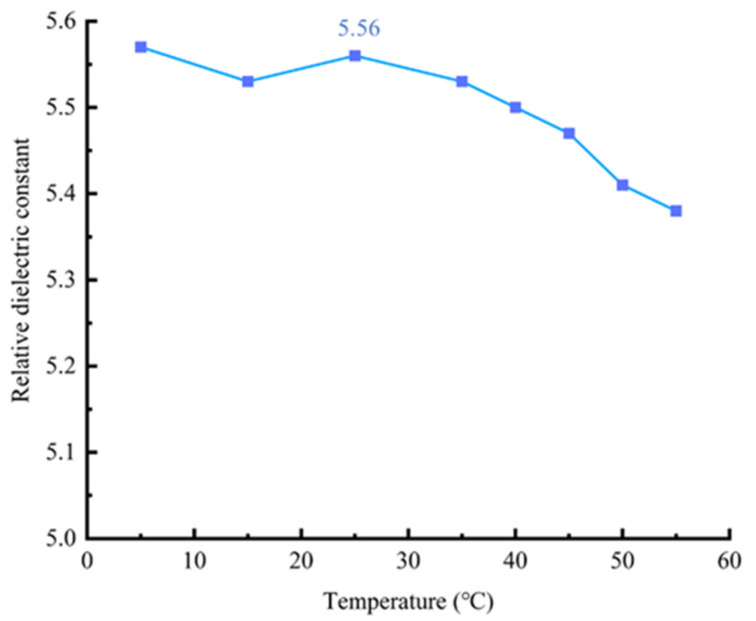
The curve of relative dielectric constant versus temperature.

We analyzed the variation in the relative permittivity of the Cu/CB/PDMS composite dielectric layer at different temperatures of 5 °C, 15 °C, 25 °C, 35 °C, 45 °C, and 55 °C. We found that when the temperature exceeds 25°C, the relative permittivity of the composite dielectric layer decreases with the increase in temperature, as shown in Figure 13. When the temperature reaches 55 °C, the relative permittivity of the composite dielectric layer decreases to 5.38, which is a reduction of approximately 3.24% compared to the value of 5.56 at 25 °C. This is because as the temperature rises, molecular motion intensifies, leading to weaker interactions between the molecules, which in turn affects the material’s polarization ability, ultimately resulting in a decrease in the permittivity.

**Figure 14:**
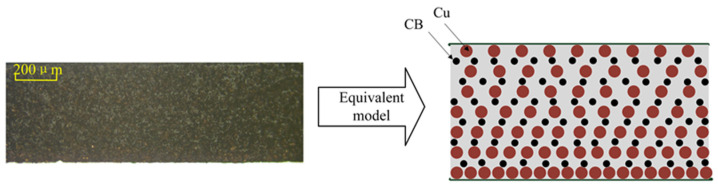
Equivalent models of Cu/CB/PDMS composite.

The cross-sectional diagram and equivalent model of the composite dielectric layer are shown in Figure 14.

### 3.3. Assembly of Sensors

**Figure 15:**
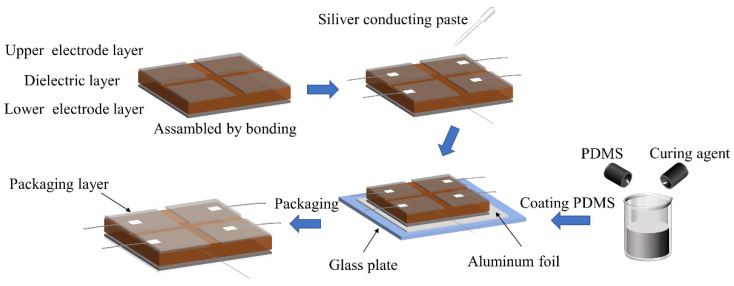
Sensor assembly process of Cu/PDMS composite sensors.

**Figure 16:**
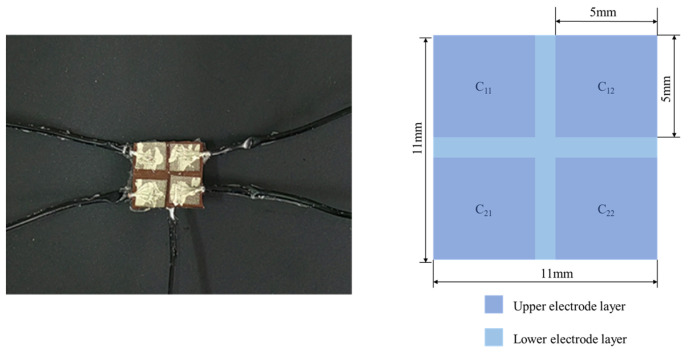
Physical diagram of the array sensor.

First, cut the conductive fabric into four 5 mm × 5 mm upper electrodes and one 11 mm × 11 mm lower electrode. Then, trim the pre-prepared dielectric layer to a size of 11 mm × 11 mm × 1 mm. Following that, paste the conductive fabric on the surface of the dielectric layer in an array pattern to serve as electrodes, and use conductive silver glue to fix the shielding wires to each electrode. After natural curing at room temperature, apply a layer of PDMS on the surface to encapsulate the entire structure. The entire assembly process is shown in Figure 15. Finally, the fabricated sensor array is shown in Figure 16, where the role of the tin foil is to facilitate demolding, making it easy to remove the finished product.

## 4. Verification of Sensor Structure Simulation

### 4.1. Tensile Testing of Materials

**Figure 17:**
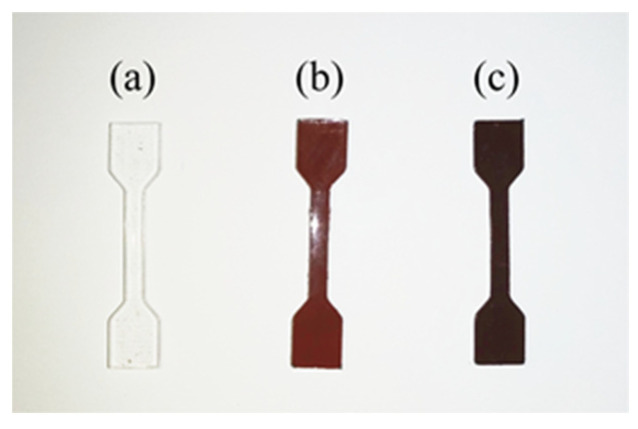
Three test samples. (**a**) PDMS materials; (**b**) Cu/PDMS composites; (**c**) CB/Cu/PDMS composite materials.

To investigate the impact of the incorporation of copper (Cu) and carbon black (CB) particles on the performance of PDMS-based composite materials, a series of uniaxial tensile tests were designed. The experiments conducted stress–strain tests on pure PDMS material, Cu/PDMS composite material, and CB/Cu/PDMS composite material. As shown in Figure 17, the mold for the test samples was designed using SolidWorks2020 software, and the dimensions were determined according to the national standard for determining the tensile stress–strain relationship of vulcanized rubber and thermoplastic rubber. The standard recommends the use of Type 2 dumbbell-shaped samples, with a specific size of 25 mm × 4 mm × 2 mm for the tensile test section, equipped with special gripping ends for fixation. The sample molds were manufactured using 3D printing technology, and then the corresponding materials were poured into the molds for preparation.

**Figure 18:**
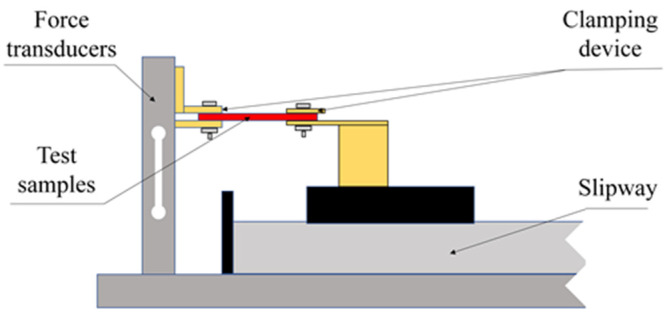
Schematic diagram of the experimental platform.

The PDMS test samples are placed on the clamps of the tensile testing platform device and tightened, as shown in Figure 18. A stepper motor driver (TB6600, Pufeide Co., Ltd., Wenzhou, China) is used to control the uniform movement of one side of the clamp on the sliding table to achieve the tensile test of the samples. The tensile distance is set according to the properties of the material. If the PDMS material is broken during the test, the data before breaking can be taken. Real-time information on the displacement of the servo motor can be obtained through the motion control card, and real-time information on the load force of the force sensor can be obtained through the data acquisition card. By controlling the system with an upper computer and using LabVIEW2014 to process the data collected by the motion control card and the data collected by the data acquisition card, the relationship between displacement and force during the stretching process can be obtained. The strain ε of the material is calculated by the ratio of the tensile change Δ*l* to the initial length *l*_0_, and the stress *σ* is calculated by the ratio of the force *F* applied to the material to the real-time cross-sectional area *S* of the material.

In this study, the tensile testing experiment used PDMS specimens with an initial cross-sectional area of 4 mm^2^ and an initial length of 25 mm. Based on the principle of volume constancy (i.e., the product of the cross-sectional area and length of the specimen remains constant during stretching), we calculated the relationship between stress and strain. The corresponding stress–strain curve is shown in Figure 18.

**Figure 19:**
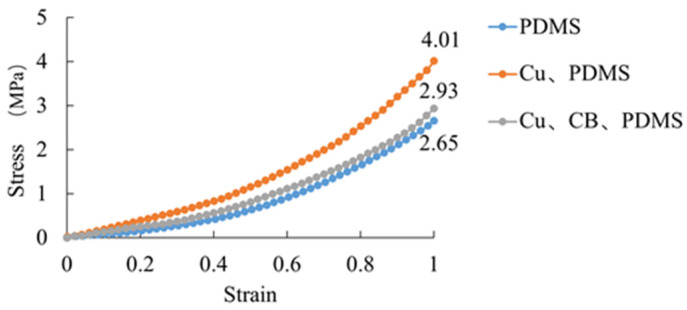
Stress–strain curves for PDMS, Cu/PDMS, and Cu/CB/PDMS composite material.

The data obtained from the tensile test can be used for subsequent finite element simulations. Since PDMS is a hyperelastic material, the stress–strain curve can be used as a parameter for PDMS material. From Figure 19, it can be seen that the stress value of pure PDMS material at a strain of one is 2.65 MPa. In contrast, the stresses of the Cu/PDMS composite material and CB/Cu/PDMS composite material at the same strain reach 4.01 MPa and 2.93 MPa, respectively. This result indicates that the incorporation of Cu and CB can significantly increase the stress required by the material under the same strain; further incorporation of CB into the Cu/PDMS composite material will moderately reduce the stress requirement.

### 4.2. Simulation of Sensor Dielectric Layer Under Pressure

PDMS is a hyperelastic material with significant nonlinear behavior. The behavior of hyperelastic materials can be described by constitutive models for large elastic deformations, including common models such as the Yeoh model, neo-Hooke model, Mooney–Rivlin model, and the Ogden model. Among them, the Yeoh model is widely used because it can accurately predict the behavior of materials under uniaxial compression. This paper selects the Yeoh model to perform finite element simulation of PDMS material, aiming to investigate its mechanical response under various loading conditions.

**Figure 20:**
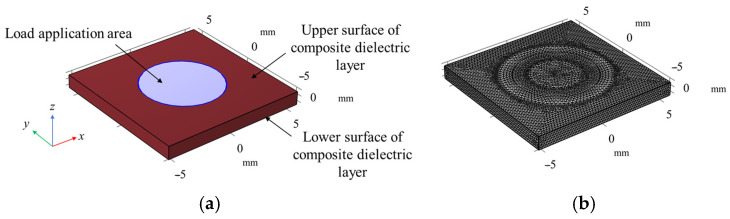
Sensor compression simulation model. (**a**) Schematic diagram of dielectric layer simulation mode; (**b**) schematic diagram of grid division.

**Figure 21:**
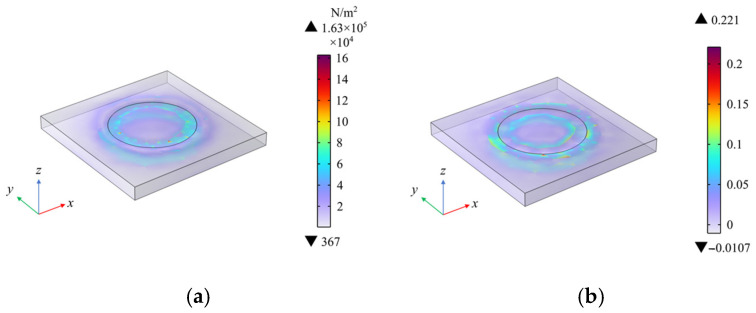
Simulation results under central area loading. (**a**) Stress distribution results; (**b**) strain distribution results.

The flexible sensor designed was subjected to compression simulation analysis using the multi-physics finite element simulation software. Since the upper and lower electrode layers are made of conductive fabric and their thickness is only 0.1 mm, they do not participate in deformation; therefore, the impact of the electrode layers is neglected in the simulation, focusing on the deformation of the dielectric layer. Figure 20 shows the simulation model and mesh division of the sensor’s dielectric layer. The specific steps of the simulation process are as follows:

A uniform load of 0.1 MPa with a radius of 3 mm was applied to the central area of the dielectric layer. The simulation results, as shown in Figure 21, display both the stress and strain contour plots. Through a comprehensive analysis of the stress and strain contour plots, it can be observed that the stress and strain distributions in the dielectric layer are relatively uniform. Although some stress concentration is observed in the circular edge areas, the overall mechanical response remains relatively stable. This uniform mechanical response provides a solid foundation for the stability of the sensor, ensuring reliable pressure sensing and response during subsequent detection processes.

**Figure 22:**
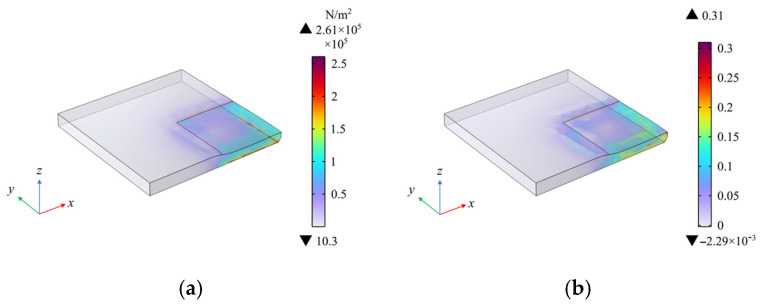
Simulation results under local area loading. (**a**) Stress distribution results; (**b**) strain distribution results.

A localized uniform load of 0.1 MPa, with a size of 5 mm × 5 mm, was applied to the bottom right corner of the dielectric layer. The simulation results are shown in Figure 22, which displays the stress and strain contour plots. Analysis of the stress and strain contour plots reveals that under the influence of the localized load, the dielectric layer surface in the area where the load was applied experienced significant stress and strain, while the areas not subjected to the load showed minimal stress and strain, being almost unaffected. This also confirms that the sensor can effectively sense and respond to stress changes in specific areas under localized load, demonstrating the capability for localized detection.

By conducting simulation experiments on the dielectric layer of the capacitive flexible pressure sensor under different pressure conditions, not only was the efficient performance of the sensor under global and localized load applications verified but also, important theoretical support was provided for the actual sample tests to be conducted, ensuring the scientific and feasibility of the experimental scheme.

### 4.3. Simulation of Capacitance Characteristics of the Composite Composed Sensor

**Figure 23:**
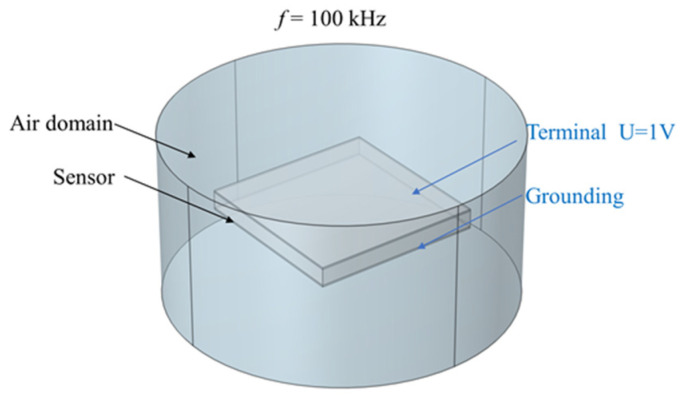
Sensor capacitance simulation model.

To explore the electrical performance of the flexible capacitive pressure sensor to be fabricated, multi-physics finite element simulation software COMSOL Multiphysics 6.0 was used to analyze the capacitive characteristics of the designed flexible sensor. In the geometry module, a three-dimensional model of the flexible capacitive sensor was constructed. A cylindrical air domain centered at the origin *o* of the spatial Cartesian coordinate system *xyz* was added to the model, with both the radius and height being 10 mm, which could effectively simulate the actual electric field distribution, thereby enhancing the accuracy of capacitance calculations. The constructed flexible capacitive sensor simulation figure is shown in Figure 23.

**Figure 24:**
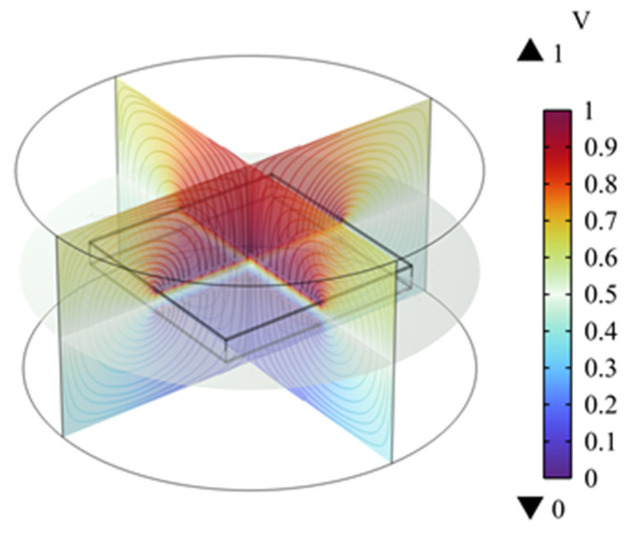
Sensor potential distribution.

The finite element calculation obtained the electric potential distribution of the flexible capacitive sensor, as shown in Figure 24. The red areas in the figure represent parts with higher electric potential, while the blue areas correspond to parts with lower electric potential.

In the post-processing stage, we extracted the admittance matrix parameters through the Global Evaluation node. The admittance matrix characterizes the relationship between current and voltage at different nodes in the system. Based on the extracted admittance matrix parameters, and in combination with Equation (8), the capacitance value of the capacitive sensor was ultimately calculated.

Capacitance can be calculated using admittance and angular frequency. The admittance of a capacitor is a complex number, where the imaginary part is related to the value of the capacitance [40]:

(8) Y=jωC

(9) C=Im(Y)ω

where Im(*Y*) represents the imaginary part of the admittance, *ω* stands for the angular frequency, and *C* denotes the capacitance value obtained from the simulation calculation. By using the global evaluation function to extract the imaginary part of the admittance as 1.8418 × 10^−6^i, the capacitance value of the capacitive sensor is found to be 2.9 pF, which is slightly higher than the experimentally measured capacitance value of pure PDMS, which is 2.78 pF. This discrepancy may arise from the use of idealized material parameters in the simulation model, such as the dielectric constant, material thickness, and area. However, in the actual manufacturing process, minor material variations such as uneven thickness or the presence of material impurities often lead to deviations between the actual capacitive characteristics and theoretical calculated values. This indicates that inevitable material variations during the manufacturing process have a significant impact on the capacitive performance of the sensor.

**Figure 25:**
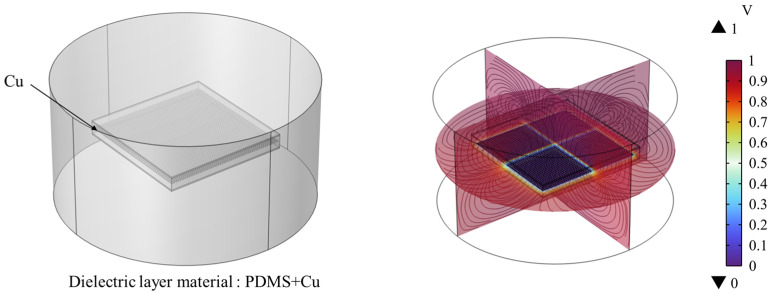
Sensor capacitance simulation model and potential distribution.

In the simulation model shown in Figure 23, conductive fillers—copper powder—were introduced. The flexible capacitive sensor simulation model is now as shown in Figure 25. To simplify the calculation process and improve simulation efficiency, the diameter of the copper powder was set to 0.1 mm, with a quantity of 2500 particles. The finite element calculation obtained the electric potential distribution of the flexible capacitive sensor under the influence of copper powder fillers. By comparing the simulation data, it can be observed that the capacitance value of the flexible capacitive sensor increased from 2.9 pF to 10.32 pF, indicating that the addition of copper powder enhanced the performance of the flexible capacitive sensor.

**Figure 26:**
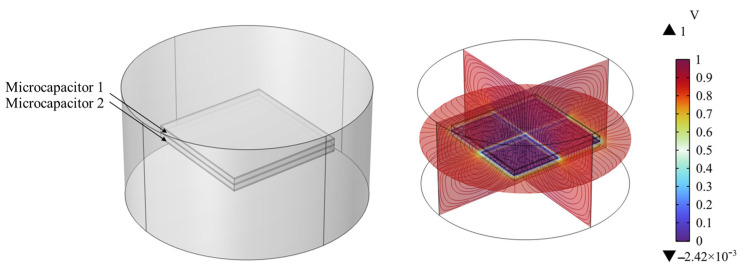
Composite dielectric layer simulation model.

To verify the composite dielectric layer model proposed in Figure 5, a composite dielectric layer simulation model as shown in Figure 26 was constructed. Compared to Figure 25, the gaps between iron powder particles in the composite dielectric layer model shown in Figure 26 are set to 0, making the flexible capacitive sensor equivalent to two micro-capacitors connected in parallel. The finite element calculation obtained the electric potential distribution of the flexible capacitive sensor under the influence of iron powder fillers. By comparing the simulation data, it can be observed that the capacitance value of the flexible capacitive sensor increased from 10.32 pF to 11.2 pF.

**Figure 27:**
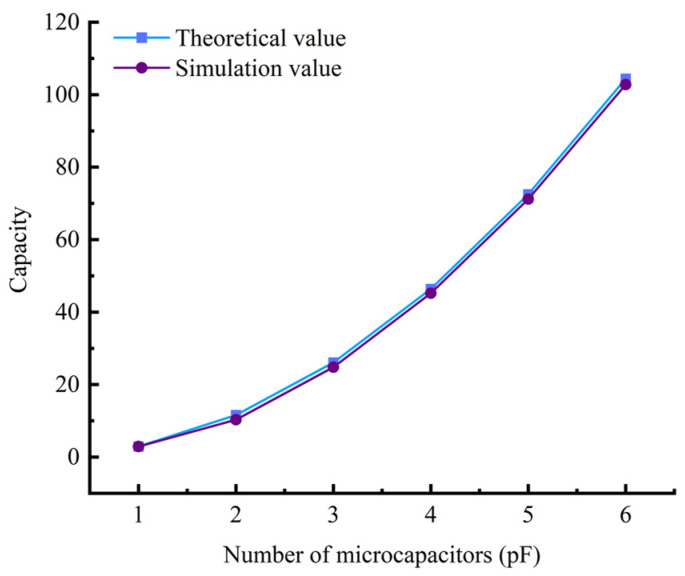
Theoretical versus simulated values for composite dielectric layer models.

Figure 27 shows a comparison between the theoretical capacitance values and the simulated capacitance values of the composite dielectric layer model. The theoretical values are calculated based on the initial capacitance value (2.9 pF) of the undoped conductive filler and are magnified according to the square of the number of parallel sensors. As can be seen from Figure 26, the simulated capacitance values have a small error compared to the theoretical capacitance values, and the capacitance value of the sensor shows a significant upward trend as the number of micro-capacitors increases. When the number of micro capacitors reaches six, the capacitance value reaches its maximum, with a simulated value of 102.8 pF. Although the trends of the theoretical and simulated values are consistent, the simulated values are generally slightly lower than the theoretical values. This may be due to the fact that factors such as differences in conductivity were not fully considered in the simulation process, leading to more conservative simulation results.

## 5. Performance Test of the Sensor

### 5.1. Output Performance Test

#### 5.1.1. Distributed Normal Force Test

**Figure 28:**
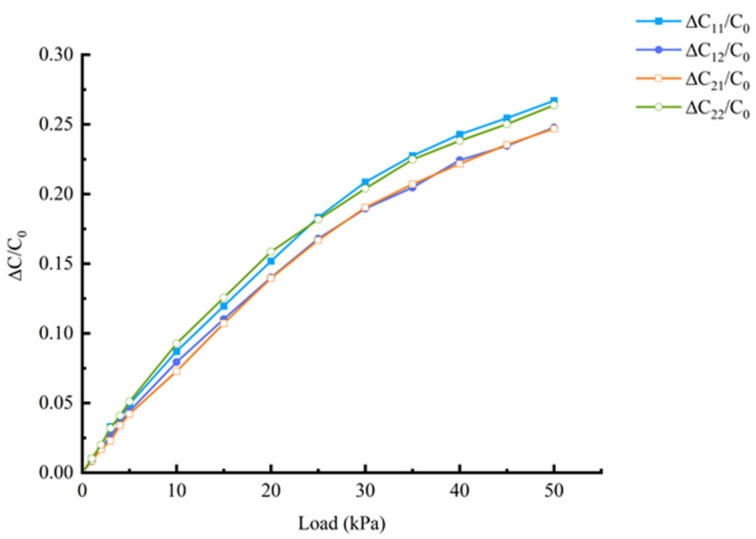
Distributed normal force output characteristic curve.

To test the response of the flexible capacitive sensor to normal force loading, a servo motor is used to control the slide stage to move in the normal direction, achieving precise force application on the sensor surface. The sensor applies and monitors the force through a digital push–pull force gauge fixed on the slide stage. The effective range of normal force is 0~50 kPa, with normal force loading at intervals of 5 kPa, and each loading point is repeated five times to ensure data stability. The capacitance value is measured using an LCR bridge, and the output capacitance values of the sensor *C*_11_, *C*_12_, *C*_21_, and *C*_22_ are recorded under different normal forces. The output characteristic curve of the sensor is shown in Figure 28, where ∆*C*/*C*_0_ represents the capacitance change rate under the corresponding load, *C*_0_ represents the initial capacitance value of the sensor, and ∆*C* is the capacitance change in the sensor when the push–pull force gauge applies a load to the flexible sensor surface in the normal force direction, with the unit being pF.

**Figure 29:**
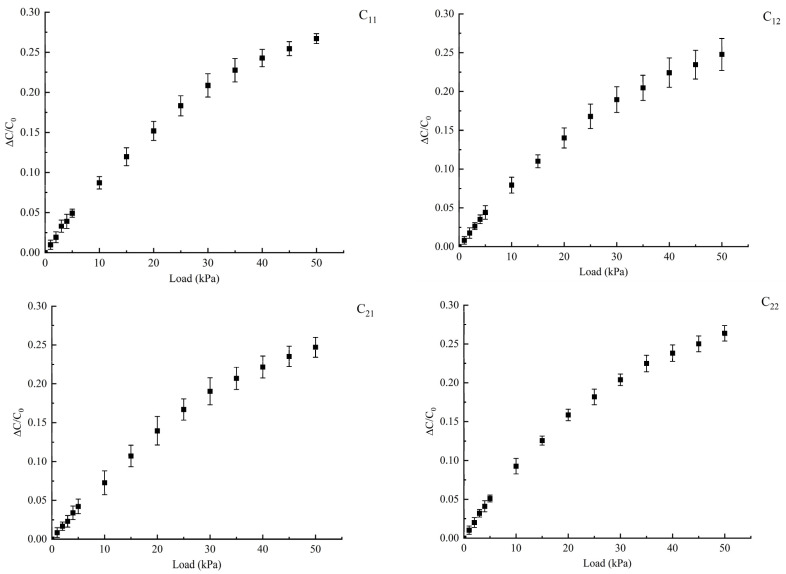
Standard deviation of the amount of change in capacitance.

From the figure, it can be observed that when the normal force is applied to the force-bearing layer of the flexible sensor, the capacitance changes of *C*_11_, *C*_12_, *C*_21_, and *C*_22_ all show an upward trend. This is due to the uniform pressure distribution generated by the upper encapsulation layer, which causes the dielectric layer to undergo corresponding deformation. Due to the symmetry of the sensor structure, the compression deformation of the dielectric layer is the same in the areas corresponding to the four capacitances when subjected to force, leading to a simultaneous reduction in the gap *d* between the four capacitances, thereby increasing the capacitance values. Moreover, the changes in each capacitance exhibit consistency. The measured capacitance values and standard deviations of *C*_11_, *C*_12_, *C*_21_, and *C*_22_ in the direction of the normal force are shown in Figure 29.

#### 5.1.2. Concentrated Force Test

**Figure 30:**
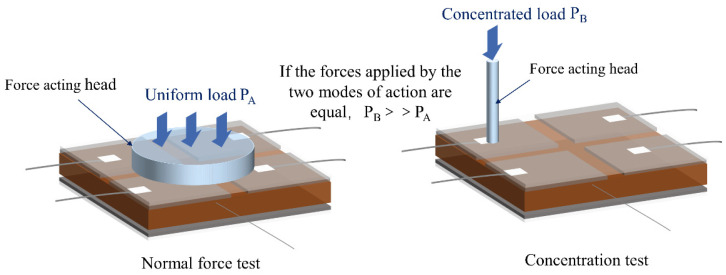
The principle of concentrated force test.

In addition, we also conducted a concentrated force test, in which the contact area between the sensor and the actuator head is a circular plane with a diameter of 1 mm. As shown in Figure 30, the concentrated force test is compared with the traditional normal force test. Although the readings of the concentrated force test are consistent with those of the normal force test, the area of action of the concentrated force is much smaller than the distribution area of the normal force. This difference leads to greater stress being applied to a local area of the sensor under the same force value, which may in turn produce a greater load response.

A high-precision LCR tester was used to test the electrical properties of the sensor. Under the action of concentrated load, a conduction phenomenon occurred between the two electrodes of the sensor, resulting in a negative capacitance value for the sensor, and the resistance value was the resistance of the electrodes and wires. Initial tests showed that when a pressure of 40 kPa was applied, the sensor first exhibited conduction. As the number of force applications to the same contact point increased, the pressure threshold for triggering conduction showed a decreasing trend. By the 20th application of force, the pressure for conduction to occur dropped to 10 kPa.

### 5.2. Sensitivity and Fatigue

The sensitivity [36] of the sensor can be expressed as the ratio of the relative change in capacitance to the change in load:

(10) $$S=\frac{\Delta C/C_{0}}{\Delta P}$$

In the formula, ∆*C* represents the change in capacitance of the sensor when a load is applied to the surface of the flexible sensor in the direction of the normal force using a push–pull force gauge, with the unit being pF, and ∆*P* represents the magnitude of the load applied during the test process, with the unit being kPa.

The sensitivity of the sensor is calculated using the mean values of the four curves in Figure 28, and the specific values can be seen in Table 1. From the table, it can be observed that the sensitivity of the sensor varies under different loading conditions. Between 0 and 10 kPa of applied force, the relative change in sensor capacitance is 0.0829, with a sensitivity of 0.00829 kPa^−1^; between 40 and 50 kPa of applied force, the relative change in capacitance is 0.0246, with a sensitivity of 0.0025 kPa^−1^.

A flexible sensor was subjected to a force of 5 kPa for 5000 cycles of pressure loading and unloading to assess its performance stability under repeated stress. The relative change in the sensor’s capacitance showed good consistency throughout the entire testing period. The test results indicated that the initial capacitance value of the sensor was 4.57 pF, which decreased to 4.31 pF after 5000 pressure cycles, with a capacitance retention rate of 94.3%. This demonstrates that the flexible sensor has good stability.

### 5.3. Repeatability

**Figure 31:**
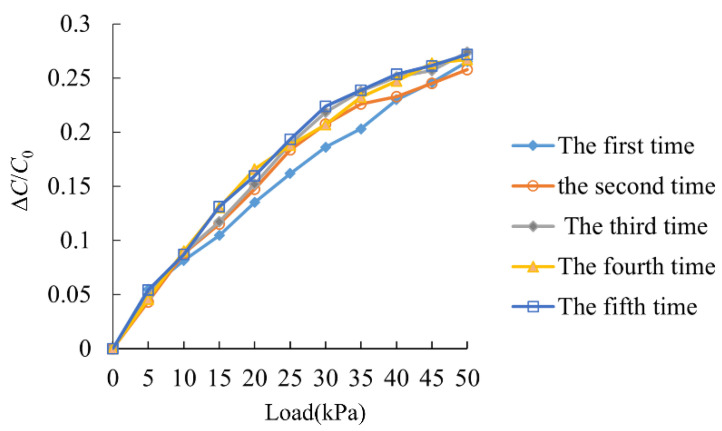
Repeatability test curve.

A normal force ranging from 0 to 50 kPa was applied to the surface of the flexible sensor, and five loading cycles were repeated under the same conditions in Figure 31. The capacitance values of the sensor during each loading cycle were measured and recorded using an LCR bridge to assess its repeatability under normal force. Sensor repeatability testing shows the capacitance variation curves during the five loading cycles, and the differences between these curves are minimal, indicating that the sensor exhibits good repeatability under normal loading.

## 6. Conclusions

This paper presents a novel capacitive array sensor based on Cu/CB/PDMS composite materials.

1. A theoretical model for the composite dielectric layer was developed, providing a detailed analysis of the mechanism by which conductive fillers (Cu powder and carbon black particles) enhance the dielectric properties of the composite material. The study reveals that when the mass fraction of conductive particles reaches the percolation threshold, they form continuous conductive paths within the dielectric layer, significantly improving its electrical performance.
2. Furthermore, the addition of carbon black particles increases the viscosity of the Cu/PDMS composite, which enhances the dispersion of Cu powder in the PDMS matrix, ultimately optimizing the dielectric properties of the material.

Through a combination of experiments and simulations, the proposed flexible sensor was validated to exhibit excellent performance in several aspects, including normal force, sensitivity, repeatability, and more. In particular, the concentrated force test revealed the sensor’s behavior under localized high-stress regions, showcasing its potential applications in complex environments. Future work will focus on optimizing the materials and structural design of the sensor to improve its sensitivity and durability while exploring its application potential in fields such as wearable devices, smart healthcare, and robotics.

## Figures and Tables

**Table 1:** The sensitivity of the sensor.

Pressure (kPa)	0~10	10~20	20~30	30~40	40~50
Relative changes (/)	0.0829	0.0646	0.0506	0.0336	0.0246
Sensitivity (kPa^−1^)	0.0083	0.0065	0.0051	0.0034	0.0025

## Data Availability

All test data mentioned in this paper will be made available upon request from the corresponding author’s email with appropriate justification.
